# Indocyanine green versus technetium‐99m with blue dye for sentinel lymph node detection in early‐stage cervical cancer: A systematic review and meta‐analysis

**DOI:** 10.1002/cnr2.1401

**Published:** 2021-05-11

**Authors:** Ilse G. T. Baeten, Jacob P. Hoogendam, Bernadette Jeremiasse, Arthur J. A. T. Braat, Wouter B. Veldhuis, Geertruida N. Jonges, Ina M. Jürgenliemk‐Schulz, Carla H. van Gils, Ronald P. Zweemer, Cornelis G. Gerestein

**Affiliations:** ^1^ Department of Gynecologic Oncology, Division of Imaging and Oncology University Medical Center Utrecht, Utrecht University Utrecht The Netherlands; ^2^ Department of Surgery Princess Máxima Center for Pediatric Oncology Utrecht The Netherlands; ^3^ Department of Radiology and Nuclear Medicine, Division of Imaging and Oncology University Medical Center Utrecht, Utrecht University Utrecht The Netherlands; ^4^ Department of Pathology, Division of Laboratory, Pharmacy and Biomedical Genetics University Medical Center Utrecht, Utrecht University Utrecht The Netherlands; ^5^ Department of Radiotherapy, Division of Imaging and Oncology University Medical Center Utrecht, Utrecht University Utrecht The Netherlands; ^6^ Julius Center for Health Sciences and Primary Care University Medical Center Utrecht, Utrecht University Utrecht The Netherlands

**Keywords:** cervical cancer, indocyanine green, meta‐analysis, sentinel lymph node, technetium‐99 m nanocolloid

## Abstract

**Background:**

The fluorescent dye indocyanine green (ICG) has emerged as a promising tracer for intraoperative detection of sentinel lymph nodes (SLNs) in early‐stage cervical cancer. Although researchers suggest the SLN detection of ICG is equal to the more conventional combined approach of a radiotracer and blue dye, no consensus has been reached.

**Aims:**

We aimed to assess the differences in overall and bilateral SLN detection rates with ICG versus the combined approach, the radiotracer technetium‐99m (^99m^Tc) with blue dye.

**Methods and Results:**

We searched MEDLINE, Embase, and the Cochrane Library from inception to January 1, 2020 and included studies reporting on a comparison of SLN detection with ICG versus ^99m^Tc with blue dye in early‐stage cervical cancer. The overall and bilateral detection rates were pooled with random‐effects meta‐analyses.

From 118 studies retrieved seven studies (one cross‐sectional; six retrospective cohorts) were included, encompassing 589 patients. No significant differences were found in the pooled overall SLN detection rate of ICG versus ^99m^Tc with blue dye. Meta‐analyses of all studies showed ICG to result in a higher bilateral SLN detection rate than ^99m^Tc with blue dye; 90.3% (95%CI, 79.8‐100.0%) with ICG versus 73.5% (95%CI, 66.4‐80.6%) with 99mTc with blue dye. This resulted in a significant and clinically relevant risk difference of 16.6% (95%CI, 5.3‐28.0%). With sensitivity analysis, the risk difference of the bilateral detection rate maintained in favor of ICG but was no longer significant (13.2%, 95%CI −0.8‐27.3%).

**Conclusion:**

ICG appears to provide higher bilateral SLN detection rates compared to ^99m^Tc with blue dye in patients with early‐stage cervical cancer. However, in adherence with the Grading of Recommendations, Assessment, Development, and Evaluation (GRADE) guidelines, the quality of evidence is too low to provide strong recommendations and directly omit the combined approach of ^99m^Tc with blue dye.

## 1 INTRODUCTION

Lymph node status is the strongest prognostic factor for survival in stage I‐II cervical cancer,^1^ highlighting the importance of nodal assessment. The sentinel lymph node (SLN) procedure is intended to reach that purpose and could play a fundamental role in reducing the need for full pelvic lymphadenectomy, thereby decreasing surgical morbidity. In addition, the subsequent frozen section examination can ascertain the lymph node status before radical uterine surgery is performed. This allows substituting radical surgery with primary chemoradiation in case of lymphatic metastases, which avoids the morbidity associated with double modality treatment.^2^, ^3^ Prerequisites for a reliable SLN procedure are a high bilateral detection rate (defined as the proportion of patients with at least one SLN detected in each hemipelvis) and low false‐negative rate (defined as tumor‐negative SLNs concurrent to tumor‐positive non‐SLNs at lymphadenectomy) to minimize the risk of undertreating cervical cancer patients.^4^, ^5^

Currently, two predominant methods for detecting SLNs in cervical cancer are in use. The first is the more conventional combination of the radiotracer technetium‐99m nanocolloid (^99m^Tc) and blue dye. The radiotracer enables preoperative imaging with SPECT‐CT, aiding in a rapid SLN identification with intraoperative detection of the radioactive signal through tissue.^6^, ^7^ Blue dye is added to visualize the afferent lymphatic architecture and SLNs during surgery, particularly those in the area near the cervix where detection with the radiotracer is hindered (ie, near the injected tracer depot). The second method uses indocyanine green (ICG), which emerged over the past decade as a new tracer for SLN detection in various cancers.^8^ ICG is visualized intraoperatively with near‐infrared (NIR) fluorescence imaging, providing real‐time visual navigation—with a better tissue penetration than blue dye.^9^, ^10^ Its feasibility has been demonstrated and early reports showed high SLN detection rates in patients with early‐stage cervical cancer.^11^, ^12^, ^13^, ^14^

It has been argued that even in the case of equivalent detection rates, the safety profile (eg, less allergic reactions than blue dye, avoidance of radioactivity) and logistics of ICG (which does not require injection in a controlled environment), will favor it over ^99m^Tc combined with blue dye.^15^, ^16^, ^17^ However, no consensus has been reached on the equivalence of detection and the implementation of ICG in SLN mapping in cervical cancer patients is not yet widely accepted. Limitations of ICG include less guidance toward unexpected SLN positions because of the absence of preoperative imaging and reduced tissue penetration compared to ^99m^Tc radioactivity.^18^

With this systematic review and meta‐analysis, we aim to assess the differences in SLN detection between ICG and the combined approach of 99mTc with blue dye in early‐stage cervical cancer patients.

## 2 MATERIALS AND METHODS

### 2.1 Systematic search

We conducted a systematic review and meta‐analysis following the Preferred Reporting Items for Systematic reviews and Meta‐Analyses (PRISMA) guideline.^19^ Before initiating our search, we drafted a protocol that contained the research question, search strategy, inclusion and exclusion criteria, quality assessment, data collection, and statistical analysis. We systematically searched the following databases:

- MEDLINE via PubMed from inception (1946) to January 1, 2021
- Embase from inception (1947) to January 1, 2021
- The Cochrane Central Register of Controlled Trials (CENTRAL; 2021, Issue 1) in the Cochrane Library

The search query combined synonyms, abbreviations, and alternative spellings for “cervical cancer,” “sentinel node biopsy,” “ICG,” and “technetium‐99m,” based on prior systematic reviews and input from a reference librarian. When the search strategy identified a conference abstract, we searched MEDLINE for an associated full‐text article by the same authors. We checked the reference lists of both the included studies and other systematic reviews on a similar topic for potentially relevant references.^15^, ^20^ All identified references were exported to the reference management program EndNote X9 (Clarivate Analytics, Philadelphia, Pennsylvania) for removal of duplicates.

### 2.2 Eligibility criteria

The search results were limited to English, Dutch, French, and German language. Peer‐reviewed studies reporting on a comparison of the overall and/or bilateral SLN detection rate of ICG versus ^99m^Tc with blue dye in patients with stage I‐II cervical cancer were included to ensure equal patient populations and surgical setting. We excluded studies that did not contain original data, conference abstracts, case reports, and editorials. The references of all included studies were cross‐checked for possible additional literature. Two reviewers (I.B., J.H.) independently selected eligible studies by first screening title and abstract, and subsequently reviewing full texts to determine their eligibility. Divergent opinions were resolved by consensus discussion. Any remaining disagreements were resolved by a third reviewer (C.G.).

### 2.3 Quality assessment

The methodological quality of all included studies was appraised independently by two reviewers (I.B., J.H.) using the validated ROBINS‐I tool.^21^ The ROBINS‐I tool assesses the risk of bias in seven domains: confounding, selection bias, classification of interventions, deviations from intended intervention, missing data, bias in measurements of outcomes, and in the selection of the reported result. The risk of bias of each domain was scored as low, moderate, serious, or critical. In adherence to the ROBINS‐I guideline, a domain classification as low risk of bias entailed a study comparable to a well‐performed randomized trial with regard to that domain. Declaring a study to be at a particular level of risk of bias for an individual domain meant that the study as a whole had a risk of bias of at least this level. When the assessed study was scored as critical, it was excluded from the analysis. Although the QUADAS‐2 tool for diagnostic accuracy studies may have been more obvious, we considered the ROBINS‐I a better fit for adequately assessing the quality of the included studies. Most of the studies retrieved with our search were cohort studies, which were particularly interested in the detection rate of both modalities (retrospectively) and not in diagnostic accuracy (sensitivity and specificity endpoints). Therefore, a flow diagram of these (non‐cross‐sectional) studies, needed for the QAUDAS‐2 tool, was not available. We have handled the retrospective cohort studies as observational intervention studies on a diagnostic intervention. In case we did include a diagnostic accuracy (ie, cross‐sectional design) study, we constructed a flow diagram and, in addition, assessed the possible risk of bias introduced by “flow and timing” of index and reference test (ie, aspects in QUADAS‐2 are scored in the “measurements of outcomes” field in the ROBINS‐I tool).

The Grading of Recommendations, Assessment, Development, and Evaluation (GRADE) guidelines were used to grade the overall quality of evidence and strength of recommendations per outcome measure.^22^ The GRADE Working Group offers four levels of evidence quality: high, moderate, low, and very low. Quality may be downgraded as a result of limitations in study design or implementation, imprecision of estimates (wide confidence intervals), variability in results, indirectness of evidence, or publication bias.^23^ Based on the quality of evidence, the strength of recommendations were formulated as strong or weak.

Differences in the quality assessment were discussed to reach a consensus between the two aforementioned reviewers. Remaining disagreements were resolved by a third reviewer (C.G.).

### 2.4 Data extraction

We developed a data extraction sheet before collecting the data. For each study, the two reviewers (IB, JH) independently collected the following data: (a) author and publication details; (b) study design; (c) study population; (d) sample size per modality (ICG versus ^99m^Tc with blue dye); (e) type of surgical approach; (f) patient and tumor characteristics (eg, age, BMI, FIGO stage); (g) technical details on the use of ICG (eg, dosage, place, and timing of injection); (h) technical details on the use of ^99m^Tc with blue dye (eg, dosage and method of preoperative imaging); (i) median/mean number of SLNs resected per patient per modality; (j) intra‐operative SLN detection rates for each modality; (k) false negatives per modality (defined as either a finding of tumor‐negative SLNs but tumor‐positive non‐SLNs with full pelvic lymphadenectomy in the same patient, or as missing a tumor‐positive SLNs due to nondetection of one modality); (l) histopathological assessment; and (m) adverse events of each modality. Studies reporting data on other malignancies in addition to cervical cancer were included and only the data on cervical cancer were extracted. Disagreements in data extraction were resolved by discussion between the two reviewers; any remaining disagreements were resolved by the third reviewer (C.G.). We contacted individual authors of studies when further clarification was desired.

### 2.5 Statistical analysis

The primary outcomes were overall and bilateral SLN detection rates per modality for ICG only or the combination of ^99m^Tc and blue dye. The overall detection rate was defined as the proportion of patients in which at least one SLN is detected and the bilateral detection rate as the proportion of patients with at least one SLN detected in each hemipelvis. The secondary outcome was the safety of both modalities, determined by the false negatives and adverse events. In the literature two definitions of false negatives are formulated. The most commonly used definition is a tumor‐negative SLN in a patient with tumor‐positive non‐SLNs on final pathology (resected during full pelvic lymphadenectomy). This can be an indication of an incorrectly identified SLN. An alternate definition of false negatives is missing a tumor‐positive SLN because it is not detected by one modality (“non‐detection”) but was detected by another modality. This definition can only be applied in cross‐sectional studies (with intrapatient comparison) and will be referred to as “false‐negative mapping.”

All analyses were performed using the statistical software R, version 4.0.0 (April 24, 2020, The R Foundation for Statistical Computing) in conjunction with the “meta” package, version 4.12‐0, created by G. Schwarzer. The overall and bilateral detection rates, with 95% confidence intervals (95%CI), were calculated from the included studies. Using a random‐effects meta‐analytical model, wherein studies are weighed based on their inverse variance (ie, more weight to studies with less variance), we calculated the pooled risk differences of the primary outcomes for both modalities. Corresponding forest plots were created. We created funnel plots, wherein standard errors are plotted against the risk differences, to visually assess the risk of selective reporting (ie, publication bias), with formal significance testing (linear regression test) only when more than 10 studies were included. Statistical significance was set at *P* < .05. When (partial) overlap in the patients of two or more of the included studies occurred, possibly over‐ or underestimating the pooled outcome, a sensitivity analysis was performed to assess the effect of excluding overlapping studies.

## 3 RESULTS

### 3.1 Literature search and risk of bias evaluation

**FIGURE 1:**
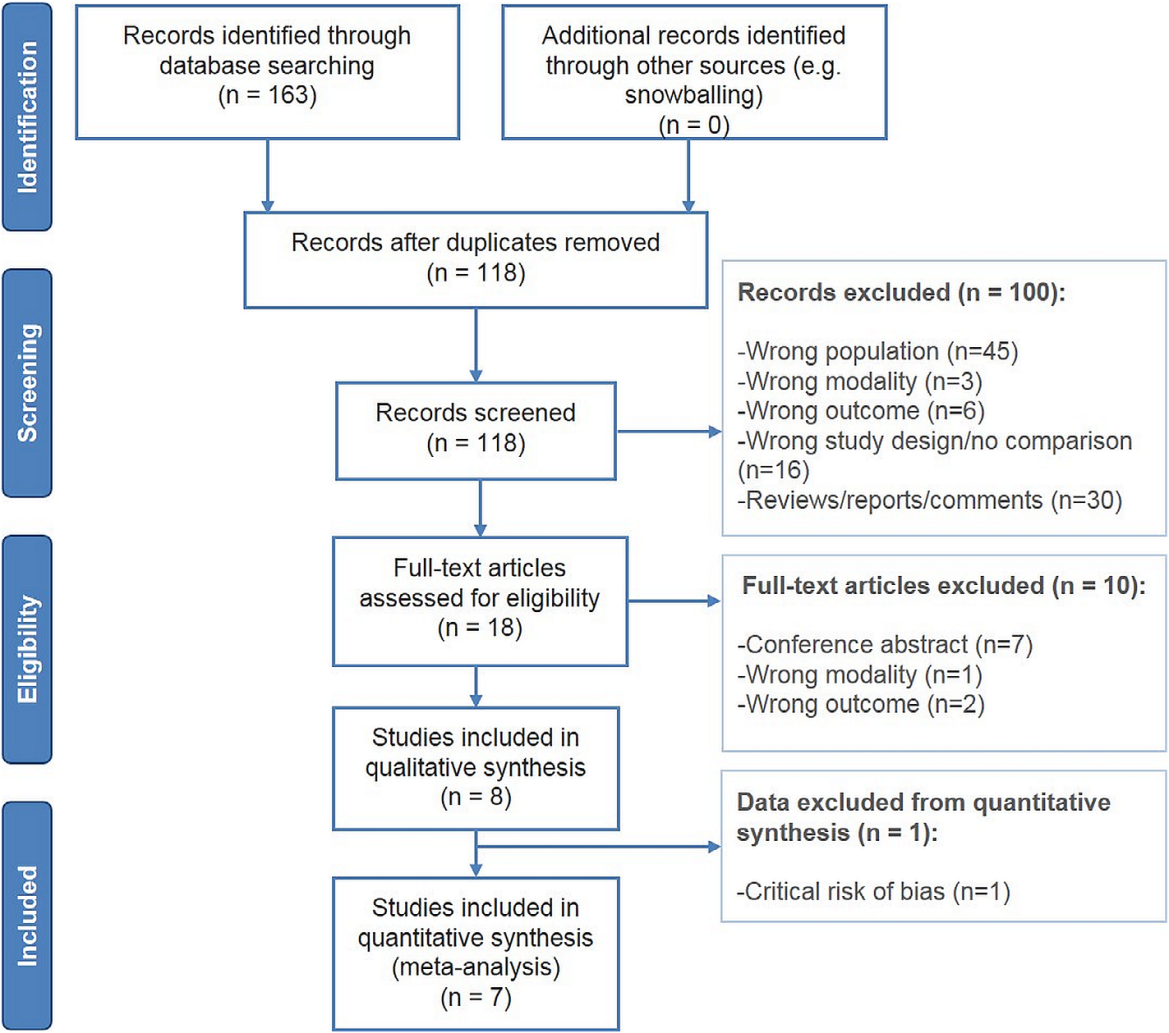
PRISMA flow chart

Figure 1 shows an overview of the systematic literature search and study selection. Our search yielded 163 publications (for the complete search see Appendix S1). After the removal of duplicates, the titles and abstracts of 118 unique articles were screened. In total, 18 articles remained for full‐text screening of which eight English‐language articles were eligible for inclusion; six retrospective cohort studies, one prospective cohort study, and one cross‐sectional study, all with consecutive patient enrolment.^24^, ^25^, ^26^, ^27^, ^28^, ^29^, ^30^, ^31^ Of the 10 excluded articles, seven were conference abstracts with no full text available, two did not present detection rates per modality (“wrong outcome”) and one did not use a combination of ^99m^Tc with blue dye (“wrong modality”). The references cited in the included eight articles were cross‐checked and did not yield any additional eligible studies.

Using the ROBINS‐I tool, the overall risk of bias was judged as “serious” in six studies and “moderate” in one study. One study had two domains judged as a “critical” risk, automatically leading to an overall “critical” risk of bias and exclusion from the analysis (Figure S1).^30^ Common risk‐increasing aspects were an unclear selection of patients, variation in patient groups, or methods of inclusion centers (within cohort studies), and the impossible blinding of surgeons assessing the outcome of both modalities/tracer groups (in the cross‐sectional study).

### 3.2 Study characteristics

The remaining seven studies included 589 patients (Table 1). Six studies exclusively investigated patients with early‐stage cervical cancer; one study investigated a combination of endometrial cancer and cervical cancer patients.^24^ Two studies did not provide baseline characteristics of cervical cancer patients per modality.^24^, ^29^

**TABLE 1:** Study characteristics and outcomes

First author, year	Study design	Study population	Inclusion period	Patients, n		Overall detection rate^a^, n (%)		Bilateral detection rate^b^, n (%)	
				ICG group	99mTc + BD group	ICG group	99mTc + BD group	ICG group	99mTc + BD group
Buda, 2016 (1)	RetrospectiveCase‐control	Cervical cancer, FIGO stage IA2 ‐IB1(single center^c^)	October 2010‐May 2015	9	28	9 (100)	28 (100)	9 (100)	23 (82.1)
Buda, 2018	RetrospectiveCase‐control	Cervical cancer after previous conisation, FIGO stage IA‐IB1 (two centers^c^)	March 2011‐April 2017	42	23	42 (100)	22 (95.7)	40 (95.2)	16 (69.6)
Buda, 2016 (2)	RetrospectiveCase‐control	Cervical cancer, FIGO stage IA2‐IB1 (five centers^c^)	2008‐2015	68	76	68 (100)	73 (96.1)	67 (98.5)	58 (76.3)
Di Martino, 2017	RetrospectiveCase‐control	Cervical cancer with tumor >2 cm, FIGO stage IB1 (four centers^c^)	2008‐2016	48	47	48 (100)	43 (91.5)	44 (91.7)	31 (66.0)
Imboden, 2015	RetrospectiveCase‐control	Cervical cancer, FIGO stage IA1 (with LVSI)‐IIB (single center^c^)	April 2008–August 2012	22^d^	36^e^	21 (95.5)	30 (83.0)	21 (95.5)	22 (61.0)
Salvo, 2017	RetrospectiveCase‐control	Cervical cancer, FIGO stage IA1‐IB1 and IIA1 (single center)	August 1997‐October 2015	56	101	50 (89.2)	94 (93.1)	32 (57.1)	68 (67.3)
Snyman, 2018	ProspectiveCross‐sectional	Cervical cancer, FIGO stage IA1‐IIA (single center)	NA	44^f^	SI	7 (87.5)	33 (91.7)	NA	NA
Soergel, 2018	Prospective Cross‐sectional	Cervical cancer, FIGO stage IA‐IIB (single center)	May 2015‐March 2017	33^g^	SI	32 (97.0)	33 (100)^h^	30 (90.9)	29 (87.9)^h^
							**With SPECT‐CT:** 25/28 (89.2)		**With SPECT‐CT:** 15/28 (53.6)

Abbreviations: 99mTc, Technetium‐99m nanocolloid; BD, blue dye; ICG, indocyanine green; LSG, lymphoscintigraphy; NA, not available; SI, same as intervention group; SLN, sentinel lymph node; SPECT‐CT, single‐photon emission computed tomography‐computed tomography.

^a^ Overall detection rate is defined as the percentage of patients in which at least one SLN is detected.

^b^ Bilateral detection rate is defined as the percentage of patients in which at least one SLN is detected in each hemipelvis.

^c^ Possible partial overlap between study populations of the studies marked with a “c.”

^d^ First seven patients also received 99mTc (“transition period”).

^e^ Five patients did not receive blue dye due to unclear reasons.

^f^ Different tracer combinations due to unclear reasons, study is excluded from analysis (only eight patients received ICG of which three in combination with blue dye and five in combination with blue dye and 99mTc).

^g^ Nine patients did not receive blue dye due to unclear reasons.

^h^ Blue dye did not identify additional SLNs that were not identified by 99mTc.

The majority, 67.1%, of the SLN procedures were performed by conventional laparoscopy. In the rest of the cases, the surgical method was not specified (Table S1). A total of 245 patients (41.6%) received fluorescent ICG during the SLN procedure, 311 patients (52.8%) received ^99m^Tc with blue dye, and 33 patients (5.6%) received ICG in adjunct to ^99m^Tc with blue dye (cross‐sectional study with intra‐patient comparison). In the 33 patients receiving both modalities, the SLNs were identified with ICG first, followed by identification with ^99m^Tc and blue dye. In this cross‐sectional study deviations from the intended protocol existed as in nine (out of 33) patients no blue dye was administered due to unclear reasons.^31^ In one cohort study not all patients in the “^99m^Tc with blue dye group” received blue dye due to unclear reasons (Table 1).^28^ Also, during the transition period from the conventional approach with ^99m^Tc with blue dye to ICG, seven patients in the “ICG group” received ^99m^Tc as well. These seven cases could not be identified separately.^28^

The methods of tracer injection and histopathological assessment of the SLNs varied among the included studies. The method (dosage and timing) of ICG injection was fairly equal, but the method of blue dye and particularly ^99m^Tc injection differed (Table S1). Three of the seven studies did not perform (or describe) a routinely frozen section analysis of the SLNs.^24^, ^26^, ^29^ Postoperative pathological assessment of the resected SLNs took place in all studies. Six studies described ultrastaging (multiple serial sectioning and immunohistochemical assessment of the SLN) being part of their pathological protocol.^24^, ^25^, ^26^, ^27^, ^28^, ^29^ In addition to the SLN procedure, all patients received a full pelvic lymphadenectomy with postoperative pathological assessment. Further details on the methods of the SLN procedure are provided in Table S1. Pictures of an intraoperative SLN procedure with ICG are shown in Figure S2.

### 3.3 SLN detection

**FIGURE 2:**
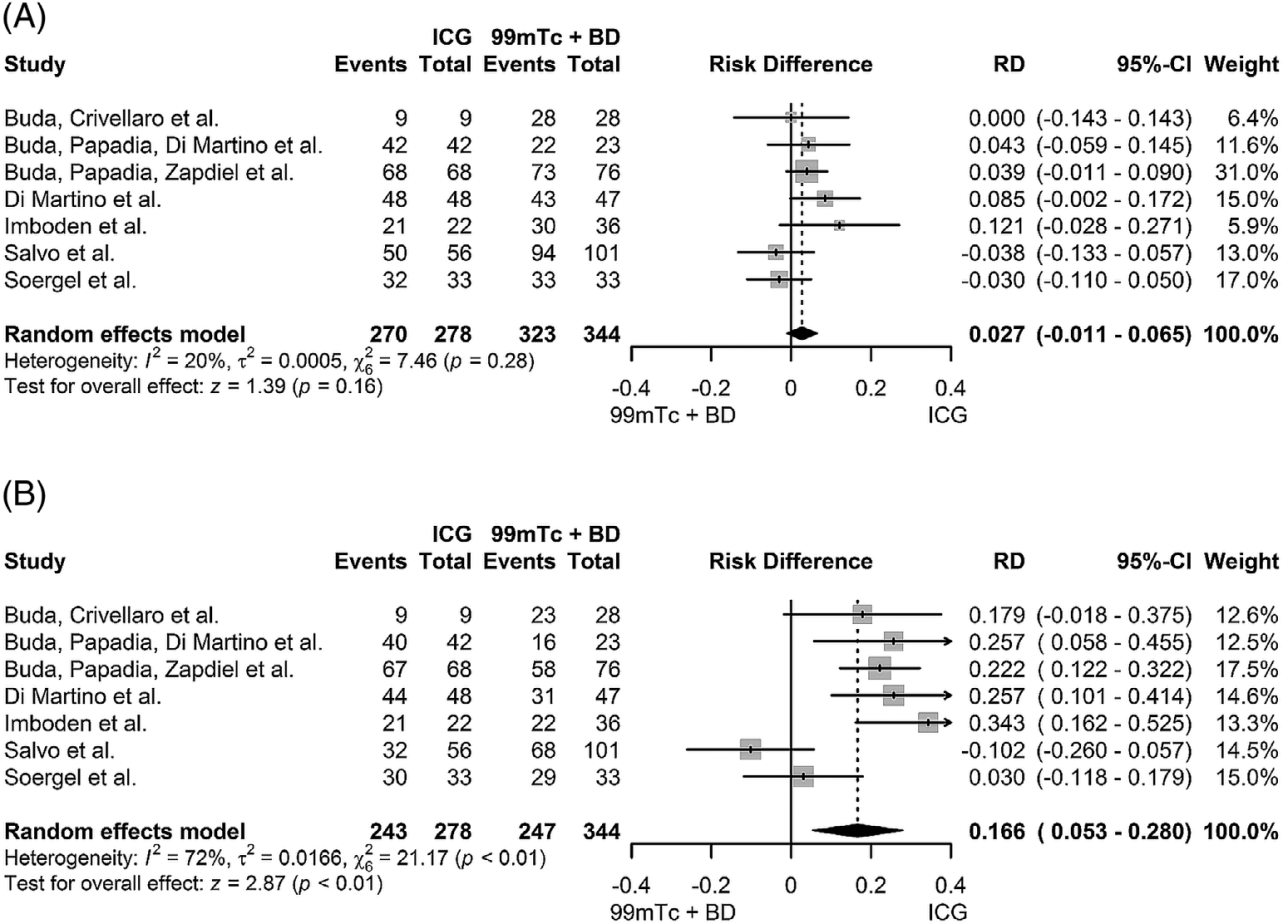
Forest plots primary analysis. Pooled risk differences in overall SLN detection (**A**) and bilateral SLN detection (**B**) of ICG versus 99mTc with blue dye (BD)

**FIGURE 3:**
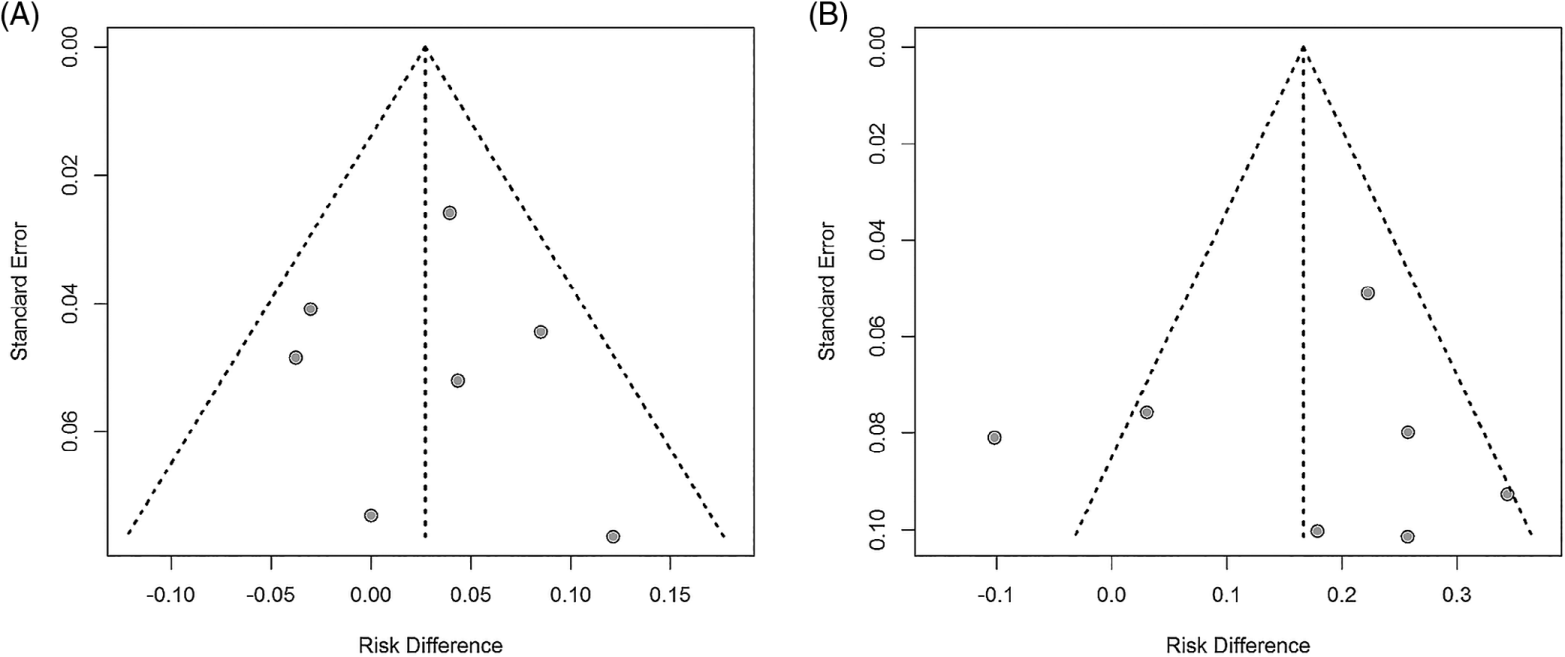
Funnel plots of overall SLN detection (**A**) and bilateral SLN detection (**B**)

The overall and bilateral SLN detection rate could be assessed in all seven studies (589 patients). The overall detection rate of ICG and ^99m^Tc with blue dye showed a pooled proportion of 98.7% (95%CI 96.5‐100.0%) and 95.6% (95%CI 92.1‐99.2%), respectively (Figure S3). No significant difference was detected upon direct comparison, with a risk difference of 2.7% (95%CI −1.1‐6.5%, *P =* .16) (Figure 2A). The pooled bilateral detection showed to be higher in the ICG group: 90.3% (95%CI 79.8‐100.0%) versus 73.5 (95%CI 66.4‐80.6%) (Figure S4), with a significant risk difference of 16.6% (95%CI 5.3‐28.0%, *P* < .01) (Figure 2B). Visual assessment of the funnel plots of both the overall and bilateral detection rate showed no convincing skewed distribution (Figure 3).

**FIGURE 4:**
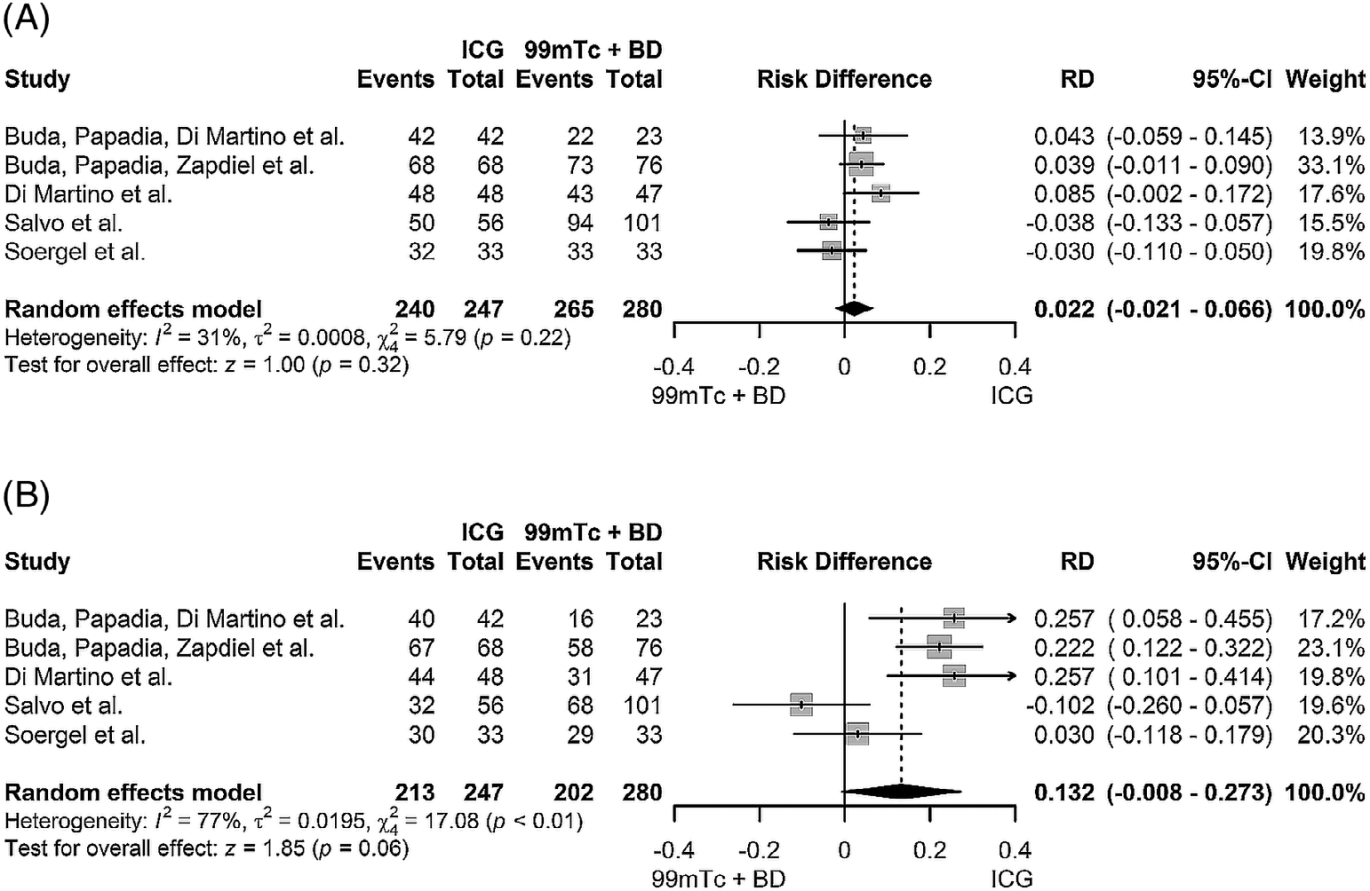
Forest plots sensitivity analysis. Pooled risk differences in overall SLN detection (**A**) and bilateral SLN detection (**B**) of ICG versus 99mTc with blue dye (BD)

Due to partial overlap of the patient populations from five studies^24^, ^25^, ^26^, ^27^, ^28^, a sensitivity analysis was performed, excluding the populations with (assumed) 100% overlap (ie, two monocenter studies, of which the cohort was later included in a multicenter study, were excluded).^24^, ^28^ The pooled overall detection rate of ICG and ^99m^Tc with blue dye did not differ substantially: 98.4% (95%CI 95.2‐100.0%) and 96.0% (95%CI 93.0‐98.9%), respectively (Figure S5). Nor did the risk difference of 2.2% (95%CI −2.1‐6.6%, *P* = .32) (Figure 4A). Although not significant, the higher bilateral detection was maintained at 87.4% (95%CI 73.1‐100.0%) in the ICG group compared with 73.9% (95%CI, 65.9‐81.9%) in the ^99m^Tc with blue dye group (Figure S6 *)*, with a risk difference of 13.2% (95%CI −0.8‐27.3%, *P =* .06)(Figure 4B *)*.

Table S1 shows the median number of SLNs detected per modality. In all studies the use of ICG yielded a higher number of SLNs, with a maximum of 26 nodes in one patient^31^ indicating these are not likely to be all SLNs but rather second echelon nodes.

### 3.4 Safety of the modalities

Six studies presented information on false negative SLNs (Table 2). Three studies reported the absence of false‐negative SLNs in both modalities (ICG versus ^99m^Tc with blue dye).^24^, ^25^, ^28^ One study reported a false negative SLN in one patient but did not specify the tracer used.^26^ One study reported false‐negative SLNs in three cases in the ICG group versus zero false‐negative cases in the ^99m^Tc with blue dye group.^27^ One patient had three false‐negative SLNs (two in the right pelvis and one in the left pelvis), detected by ICG as well as ^99m^Tc with blue dye (intrapatient comparison), with tumor‐positive non‐SLNs in final pathology.^31^

**TABLE 2:** Safety of the modalities

Author, year	False‐negative rate^a^		Adverse events	
	ICG	99mTc + BD	ICG	99mTc + BD
Buda, 2016 (1)	0/NA	0/NA	NA	NA
Buda, 2018	0/6	0/2	NA	NA
Buda, 2016 (2)	NA/10^b^	NA/17^b^	NA	NA
Di Martino, 2017	3/13	0/13	NA	NA
Imboden, 2015	0/5	0/9	NA	NA
Salvo, 2017	NA	NA	NA	NA
Soergel, 2018	1/8	1/8	0	NA

Abbreviations: 99mTc, Technetium‐99 m nanocolloid; BD, blue dye; ICG, indocyanine green; NA, not available.

^a^ Patients with false‐negative SLN (a metastatic non‐SLN and a negative SLN)/all cases with tumor‐positive lymph nodes in final pathology.

^b^ One false‐negative SLN overall, not clear in which group.

False‐negative mapping (using the alternate definition) occurred in one case (out of 33 patients).^31^ In this case ^99m^Tc with blue dye resulted in unilateral SLN detection and consequently missed a tumor‐positive SLN on the contralateral side, which was identified by ICG. In none of the cases, any ICG negative but ^99m^Tc or blue dye positive SLN showed tumor infiltration. Due to the limited data, the false negatives (using both definitions) could not be reliably pooled. None of the included studies reported adverse events of ICG, ^99m^Tc or blue dye.

### 3.5 GRADE assessment

The quality of evidence for both the outcome measures overall and bilateral detection rate was scored as very low due to the inclusion of mainly observational cohort studies (considered low quality), of which several studies with a high risk of bias (criterion for downgrading to very low quality). In addition, the pooled proportion of bilateral detection showed a relatively high heterogeneity (*I* ^2^ > 50%) and potential of publication bias.

## 4 DISCUSSION

With this systematic review, we assessed the clinical evidence of seven low‐quality studies, all comparing the SLN detection of the two modalities (ICG versus ^99m^Tc with blue dye), by including nearly 600 patients in a meta‐analysis. Our results showed the overall detection rate of both modalities was high and not significantly different. A clinically relevant and significant risk difference in bilateral detection rate was observed of almost 17% in favor of ICG. Due to the limited data provided in the included studies, it was not possible to accurately compare the safety (in terms of false negatives and adverse events) of both modalities. The risk of bias in the included studies was substantial due to the (mainly) retrospective cohort study designs and the inability to blind the surgeons to the detection of different tracers in the cross‐sectional study. Therefore, we were not able to strongly recommend one approach for detecting SLNs over the other.

The results of this systematic review match those of previous studies. A meta‐analysis in a combination of endometrial and cervical cancer patients showed higher bilateral detection rates with ICG compared to a ^99m^Tc with blue dye, albeit not significantly.^15^ Another comprehensive systematic review and meta‐analysis on ICG versus conventional tracers in multiple malignancies reported a significantly higher bilateral detection rate with ICG compared to ^99m^Tc with blue dye, specifically in gynecological cancers.^32^ The recent prospective study by Lührs et al showed an impressive higher bilateral SLN detection with ICG compared to ^99m^Tc alone in patients with cervical cancer: 98.5% with ICG versus 60% with ^99^mTc.^33^ However, in this study, no preoperative imaging was performed and no blue dye was added to the procedure, both of which have shown to increase the bilateral SLN detection rate.^34^, ^35^, ^36^

The underlying hypothesis of a higher bilateral SLN detection with ICG versus the conventional tracers, especially ^99m^Tc, is not yet clarified. It is suggested that ICG provides a better tissue penetration than blue dye, which makes ICG easier to identify for the surgeon, and previous relatively large studies in endometrial cancer substantiated these suggestions by showing a higher SLN detection rate of ICG compared to blue dye alone.^16^, ^37^ Besides detection rates, other considerations and clinical aspects of ICG over ^99m^Tc with blue dye merit further explanation. Using ICG is potentially cheaper (ie, once a fluorescence scope has been purchased), not painful since it is intraoperatively injected and logistically less challenging since the injection does not require a radiation‐safe environment. Rare adverse events have been reported with ICG^38^, ^39^ which also applies to ^99m^Tc nanocolloid.^40^ Blue dye, on the other hand, has a considerable documented risk of allergic reactions, including anaphylactic shock.^41^ Also, ICG offers the possibility of intra‐operative imaging and real‐time guiding the surgeon toward the SLN. The advantage of visualizing the afferent lymph vessels further facilitates identifying the correct SLN and possibly decreases surgical morbidity.

Nevertheless, ICG to has its disadvantages. The FILM trial, comparing ICG with blue dye only, reported a higher occurrence of presumed SLNs with a bright signal on NIR fluorescence imaging that was not confirmed to be nodes on final pathology but only lymphatic trunks or adipose tissue.^37^ This is an important finding since failing to excise the true SLN could result in missed lymph node metastases (false negative). In addition, the bright signal and rapid spreading of ICG (due to its small hydrodynamic diameter) may result in the excision of the second and third echelon lymph nodes. This is also demonstrated by the higher average number of SLNs when ICG is used, both by the studies included in our systematic review as by others (eg, Lührs et al showed a median of six SLNs with ICG versus three SLNs with ^99m^Tc).^33^ Besides the potential extra morbidity resulting from this, pathologic ultrastaging of the excised SLN is a time‐consuming and expensive process. Another disadvantage of ICG is the tissue penetration of NIR fluorescence imaging of approximately 1 cm^42^, lower than that of gamma rays and especially limited in patients with a high body mass index.^9^, ^11^ Following the green lymphatic vessels toward the, sometimes unexpected, SLN positions will be complicated when these lymphatic vessels are covered by a layer of more than 1 cm of fatty tissue. The biggest disadvantage of ICG is considered to be the lack of preoperative imaging.^18^ The use of preoperative imaging can guide the surgeon directly toward the SLN, which can prevent extensive disruption of the surrounding tissue (ie, retroperitoneum). Therefore, the use of radiotracers may still be advantageous, since it allows preoperative planning and intraoperative identification of deeper located SLNs.^9^ It is suggested that a hybrid tracer of ^99m^Tc linked to ICG could overcome these issues.^18^ With this hybrid tracer the lead‐time of ICG will be equal to that of ^99m^Tc, which also reduces the risk of detecting second echelon nodes.

Recent literature reported higher SLN detection rates from using ^99m^Tc with blue dye than what we found in our meta‐analysis. In the multi‐institutional prospective study on SLN procedure in early‐stage cervical cancer, the SENTICOL I study, a 97.8% overall detection rate and 76.5% bilateral detection rate were reported for SLN mapping with ^99m^Tc with blue dye.^4^ Combined data of the SENTICOL I and the subsequent SENTICOL II showed an even higher bilateral detection rate of 80.5%.^43^ Although the pooled percentage of bilateral detection of ^99m^Tc with blue dye found in this meta‐analysis was substantially lower, it is comparable to the bilateral detection rate of 72% reported in a review by Tax et al.^44^ It raises the question: is ICG alone really superior to ^99m^Tc with blue dye or do the studies included in this meta‐analysis achieve sub‐optimal results with the combined approach? One of the factors that may explain the difference in detection rates, and provide an answer to this, is the learning curve effect.

Previous literature has suggested that a learning curve of the SLN procedure exists. The learning curve of SLN mapping with ^99m^Tc with blue dye, to achieve >90% (overall) SLN detection, has been established in endometrial cancer at 30 cases per surgeon.^45^ This is in line with the recently reported learning curve of at least 27 cases for SLN mapping with ICG.^46^ A learning curve effect is further substantiated by Balaya et al, who showed that centers with less than five patients per year had significantly lower bilateral mapping rates than the so‐called “high skilled centers” (≥5 patients per year).^43^ The learning curve of the SLN procedure—irrespective of the tracer used—may have contributed to the variation in reported detection rates of ^99m^Tc with blue dye. In this meta‐analysis most studies started their SLN procedure with ^99m^Tc with blue dye before switching to ICG, potentially favoring the latter. Additionally, researchers suggest NIR fluorescence imaging has a steep learning curve, since most surgeons are trained in operating while using a monitor (in laparoscopic surgery), and practiced in the use of an additional probe.^9^

Other risk factors for failed SLN detection of ^99m^Tc with blue dye have been described. Balaya et al showed tumor size >2 cm, BMI above 30 kg/m^2^, and age above 70 years yielded lower bilateral SLN detection rates.^43^ Only one of the included studies in our meta‐analysis reported on the effect of tumor size on the detection rates.^26^ The researchers described a significantly higher bilateral SLN mapping in the ICG group in patients with a primary tumor >2 cm compared to the ^99m^Tc with blue dye group (100% (33/33) versus 64% (21/33), respectively; *P* = .001), while no significant difference existed in patients with a tumor size ≤2 cm (97% (34/35) versus 86% (37/43), respectively).^26^ None of the included studies assessed the impact of BMI, anatomical location, size or tumor‐positivity of the SLNs, or other relevant confounders for SLN detection.

A strength of this review is its sole focus on cervical cancer, as previous reports on SLN mapping mixed both cervical and endometrial cancer patients.^15^, ^20^ As the anatomical pattern of lymph draining in endometrial cancer differs from cervical cancer^47^ which could influence tracer performance, we have limited this review to cervical cancer patients only. Another strength is that we only included studies comparing the two modalities, limiting variation in methodology and case selection.

Our systematic review has limitations. Firstly, we could not rule out publication bias, which reflects the increased likelihood of a study being published when the study has a positive result, occurred. The funnel plots showed no convincing indication of larger studies with lower SE's reporting smaller benefits of ICG compared to smaller studies but, with only seven studies included, no formal significance testing could be reliably performed. As mentioned before, studies on the SLN detection rate of ^99m^Tc with blue dye (without comparison group) often reported higher bilateral detection rates than the studies included in this systematic review. Secondly, there were certain case mix and methodological differences between the studies that may have influenced the detection rates. The surgical modality in the selected studies differed from robot‐assisted laparoscopy, conventional laparoscopy, and laparotomy, all with different ICG‐NIR fluorescence platforms. Finally, the partial overlap in populations that existed between some studies could have led to overestimation of the risk differences. This effect is likely limited in view of our results from the sensitivity analysis. Leaving all or some of these studies in or out of the primary analysis could have resulted in selection bias from a review standpoint.

## 5 CONCLUSIONS

In early‐stage cervical cancer patients, the use of ICG appears to result in higher bilateral SLN detection compared to the more conventional combination of ^99m^Tc and blue dye. Given the advantages of ICG, these promising results could potentially lead to a widely adopted shift from ^99m^Tc with blue dye to the use of ICG only. However, in adherence with the GRADE approach, the quality of evidence is too low to provide strong recommendations and directly omit the combined approach of a radiotracer with blue dye. Larger prospective studies —preferably with the bilateral detection rate and false‐negative mapping as endpoints— are needed to further substantiate the superiority of ICG.

## AUTHOR CONTRIBUTIONS

**Ilse Baeten:** Conceptualization; formal analysis; investigation; methodology; visualization; writing‐original draft. **Jacob Hoogendam:** Conceptualization; formal analysis; investigation; methodology; visualization; writing‐original draft. **Bernadette Jeremiasse:** Validation; writing‐review & editing. **Arthur Braat:** Validation; writing‐review & editing. **Wouter Veldhuis:** Validation; writing‐review & editing. **Geertuida Jonges:** Validation; writing‐review & editing. **Ina Jürgenliemk‐Schulz:** Validation; writing‐review & editing. **Carla van Gils:** Methodology; validation; writing‐review & editing. **Ronald Zweemer:** Conceptualization; supervision; validation; writing‐review & editing. **Cornelis Gerestein:** Conceptualization; investigation; methodology; project administration; validation; writing‐review & editing.

## CONFLICT OF INTEREST

R.Z. is a proctor for robot‐assisted surgery in gynecological oncology on behalf of Intuitive Surgical Inc. C.v.G. reports grants and other from Bayer Pharma, all outside the submitted work. The other authors declare no conflicts of interest.

## ETHICAL STATEMENT

Not applicable.

## Supporting information


**Appendix S1.** Systematic search strings

**Figure S1.** Quality assessment using the ROBINS‐I tool

**Figure S2.** Intraoperative pictures of robot‐assisted SLN procedure with ICG on the left A, and right pelvic site B. White light inspection (upper) and NIR fluorescence light inspection (lower)

**Figure S3.** Pooled overall SLN detection of ICG A, and ^99m^Tc with blue dye B

**Figure S4.** Pooled bilateral SLN detection of ICG A, and 99mTc with blue dye B

**Figure S5.** Pooled overall SLN detection of ICG A, and 99mTc with blue dye B, in the sensitivity analysis

**Figure S6.** Pooled bilateral SLN detection of ICG A, and 99mTc with blue dye B, in the sensitivity analysis

**Table S1.** Detailed study characteristics

## Data Availability

Data sharing is not applicable to this article as no new data were created or analyzed in this study.
