# Supplementary material for: Indocyanine green versus technetium‐99m with blue dye for sentinel lymph node detection in early‐stage cervical cancer: A systematic review and meta‐analysis
Source: Cancer Rep (Hoboken). 2021 May 11;5(1):e1401. doi: 10.1002/cnr2.1401 (PMC8789613; doi:10.1002/cnr2.1401)
Supplement: Supplementary file 1 — Appendix S1. Systematic search strings [file CNR2-5-e1401-s006.docx]

**Appendix 1. Systematic search strings (conducted 01/04/2020)**

**MEDLINE via PubMed (1946 – 01/01/2021)**

(((Cervix[Title/Abstract] OR cervical[Title/Abstract] OR cervix uteri[MeSH Terms]) AND (Carcino*[Title/Abstract] OR cancer*[Title/Abstract] OR neoplas*[Title/Abstract] OR tumour*[Title/Abstract] OR tumor[Title/Abstract] OR tumors[Title/Abstract] OR Malign*[Title/Abstract] OR "Neoplasms"[Mesh])) OR uterine cervical neoplasm[MeSH Terms])

AND

(sentinel[Title/Abstract] OR SNB[Title/Abstract] OR SLN[Title/Abstract] OR SLNB[Title/Abstract] OR SLNM[Title/Abstract] OR SLND[Title/Abstract] OR lymphatic map*[Title/abstract] OR sentinel lymph node[MeSH Terms] OR sentinel lymph node biopsy[MeSH Terms])

AND

(Indocyanine green[Title/Abstract] OR Indocyanin*[Title/Abstract] OR ICG[Title/Abstract] OR IC green[Title/Abstract] OR Fluorescen*[Title/Abstract] OR near-infrared[Title/Abstract] OR NIR [Title/Abstract] OR indocyanine green[MeSH Terms] OR Fluorescence[MeSH Terms] OR Fluorescent Dyes[MeSH Terms])

AND

(Technetium[Title/Abstract] OR 99m Tc[Title/Abstract] OR 99mTc[Title/Abstract] OR Tc 99[Title/Abstract] OR Tc99[Title/Abstract] OR Tc 99m[Title/Abstract] OR Tc99m[Title/Abstract] OR Nanocolloid[Title/Abstract] OR Radiocolloid[Title/Abstract] OR Radiotracer[Title/Abstract] OR Technetium[MeSH Terms] OR Technetium Tc 99m Sulfur Colloid[MeSH Terms])

***Filter language: English/Dutch/German/French***

***Records: 39***

**Embase (1947 – 01/01/2021)**

((‘Cervix’:ti,ab,kw OR ‘cervical’:ti,ab,kw OR ‘cervix uteri’/exp) AND (‘Carcino*’:ti,ab,kw OR ‘cancer*’:ti,ab,kw OR ‘neoplas*’:ti,ab,kw OR ‘tumour’:ti,ab,kw OR ‘tumor’:ti,ab,kw OR ‘tumors’:ti,ab,kw OR ‘Malign*’:ti,ab,kw OR ‘Malignant Neoplasms’/exp)) OR ‘uterine cervix cancer’/exp

AND

‘sentinel’:ti,ab,kw OR ‘SNB’:ti,ab,kw OR ‘SLN’:ti,ab,kw OR ‘SLNB’:ti,ab,kw OR ‘SLNM’:ti,ab,kw OR ‘SLND’:ti,ab,kw OR ‘lymphatic map*’:ti,ab,kw OR ‘sentinel lymph node’/exp OR ‘sentinel lymph node biopsy’/exp

AND

‘Indocyanine green’:ti,ab,kw OR ‘Indocyanin*’:ti,ab,kw OR ‘ICG’:ti,ab,kw OR ‘IC green’:ti,ab,kw OR ‘Fluorescen*’:ti,ab,kw OR ‘near-infrared’:ti,ab,kw OR ‘NIR’:ti,ab,kw OR ‘indocyanine green’/exp OR ‘fluorescence’/exp OR ‘fluorescent dye’/exp

AND

‘Technetium’:ti,ab,kw OR ‘99m Tc’:ti,ab,kw OR ‘99mTc’:ti,ab,kw OR ‘Tc 99’:ti,ab,kw OR ‘Tc 99m’:ti,ab,kw OR ‘Tc99’:ti,ab,kw OR ‘Tc99m’:ti,ab,kw OR ‘Nanocolloid’:ti,ab,kw OR ‘Radiocolloid’:ti,ab,kw OR ‘Radiotracer’:ti,ab,kw OR ‘technetium 99m’/exp OR ‘technetium’/exp OR ‘albumin tc 99m’/exp

AND

([dutch]/lim OR [english]/lim OR [french]/lim OR [german]/lim)

***Records:*** *114*

**Cochrane Library; The Cochrane Central Register of Controlled Trials (CENTRAL; 2021, Issue 1)**

(Cervix:ti,ab,kw OR cervical:ti,ab,kw) AND (Carcino*:ti,ab,kw OR cancer*:ti,ab,kw OR neoplas*:ti,ab,kw OR tumour:ti,ab,kw OR tumor:ti,ab,kw OR tumors:ti,ab,kw OR Malign*:ti,ab,kw)

AND

sentinel:ti,ab,kw OR SNB:ti,ab,kw OR SLNB:ti,ab,kw OR SLN:ti,ab,kw OR SLNM:ti,ab,kw OR SLND:ti,ab,kw OR “lymphatic map*”:ti,ab,kw

AND

“Indocyanine green”:ti,ab,kw OR Indocyanin*:ti,ab,kw OR ICG:ti,ab,kw OR “IC green”:ti,ab,kw OR Fluorescen*:ti,ab,kw OR near-infrared:ti,ab,kw OR NIR:ti,ab,kw

AND

Technetium:ti,ab,kw OR “99m Tc”:ti,ab,kw OR 99mTc:ti,ab,kw OR “Tc 99”:ti,ab,kw OR Tc99:ti,ab,kw OR “Tc 99m”:ti,ab,kw OR Tc99m:ti,ab,kw OR Nanocolloid:ti,ab,kw OR Radiocolloid:ti,ab,kw OR Radiotracer:ti,ab,kw

***Records:*** *10*
