# Supplementary material for: Indocyanine green versus technetium‐99m with blue dye for sentinel lymph node detection in early‐stage cervical cancer: A systematic review and meta‐analysis
Source: Cancer Rep (Hoboken). 2021 May 11;5(1):e1401. doi: 10.1002/cnr2.1401 (PMC8789613; doi:10.1002/cnr2.1401)
Supplement: Supplementary file 2 — Figure S1. Quality assessment using the ROBINS‐I tool [file CNR2-5-e1401-s005.pdf]

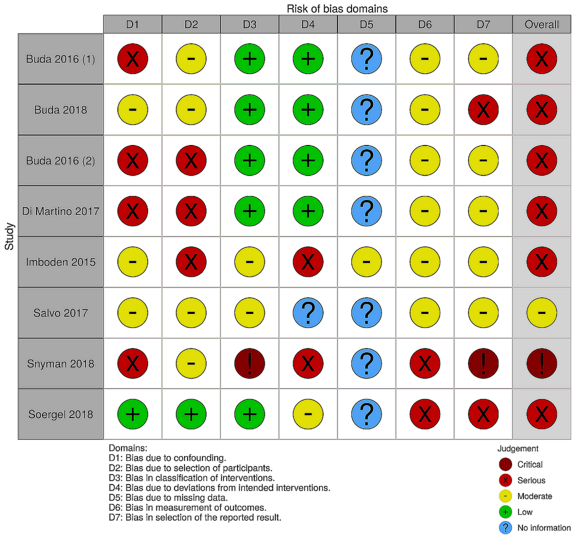
Risk of bias domains
D1
D2
D3
D4
D5
D6
D7
Overall
Study
Buda 2016 (1)
Buda 2018
Buda 2016 (2)
Di Martino 2017
Imboden 2015
Salvo 2017
Snyman 2018
Soergel 2018
Domains:
D1: Bias due to confounding.
D2: Bias due to selection of participants.
D3: Bias in classification of interventions.
D4: Bias due to deviations from intended interventions.
D5: Bias due to missing data.
D6: Bias in measurement of outcomes.
D7: Bias in selection of the reported result.
Judgement
Critical
Serious
Moderate
Low
No information
