# Supplementary figures and images for: Indocyanine green versus technetium‐99m with blue dye for sentinel lymph node detection in early‐stage cervical cancer: A systematic review and meta‐analysis
Source: Cancer Rep (Hoboken). 2021 May 11;5(1):e1401. doi: 10.1002/cnr2.1401 (PMC8789613; doi:10.1002/cnr2.1401)

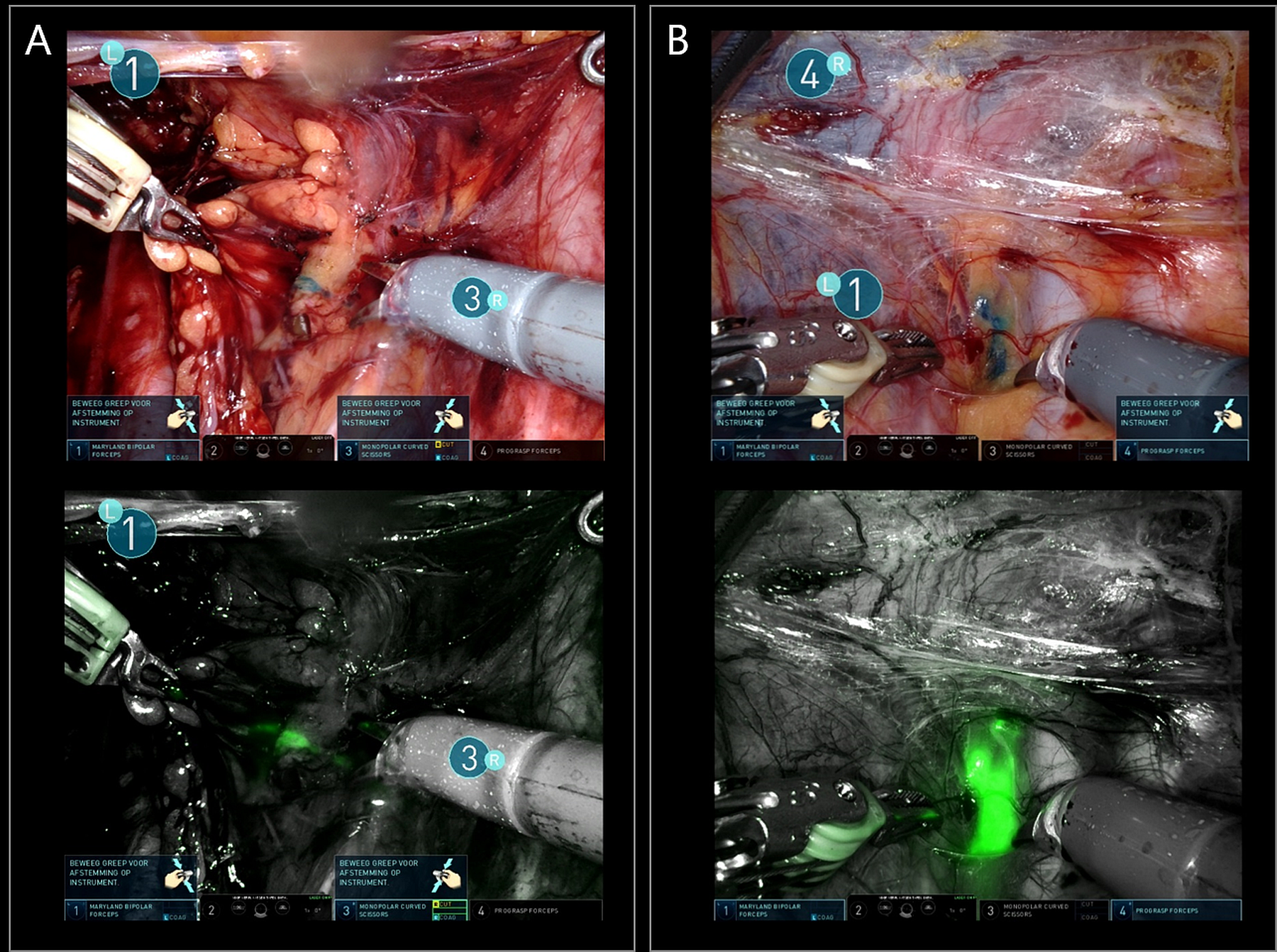

Supplement: Supplementary file 3 — Figure S2. Intraoperative pictures of robot‐assisted SLN procedure with ICG on the left A, and right pelvic site B. White light inspection (upper) and NIR fluorescence light inspection (lower) [file CNR2-5-e1401-s003.tif]

A

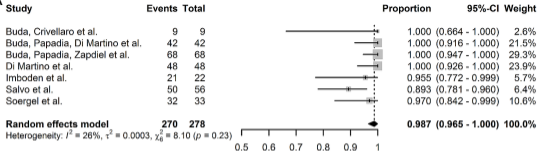

B

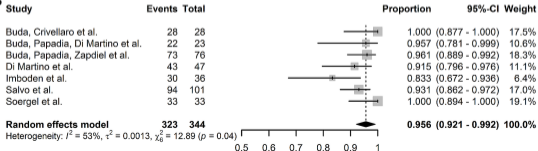

Supplement: Supplementary file 4 — Figure S3. Pooled overall SLN detection of ICG A, and 99mTc with blue dye B [file CNR2-5-e1401-s008.pdf]

A

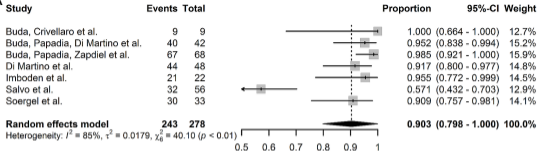

B

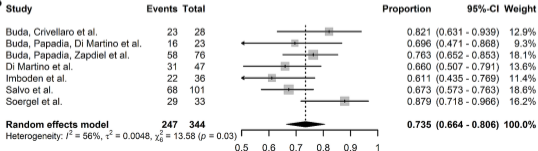

Supplement: Supplementary file 5 — Figure S4. Pooled bilateral SLN detection of ICG A, and 99mTc with blue dye B [file CNR2-5-e1401-s007.pdf]

A

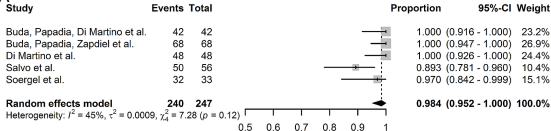

B

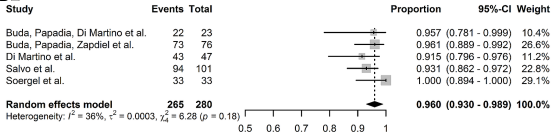

Supplement: Supplementary file 6 — Figure S5. Pooled overall SLN detection of ICG A, and 99mTc with blue dye B, in the sensitivity analysis [file CNR2-5-e1401-s001.pdf]

A

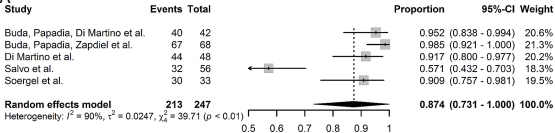

B

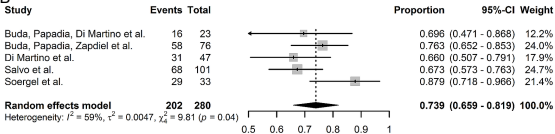

Supplement: Supplementary file 7 — Figure S6. Pooled bilateral SLN detection of ICG A, and 99mTc with blue dye B, in the sensitivity analysis [file CNR2-5-e1401-s004.pdf]
