# Supplementary material for: Indocyanine green versus technetium‐99m with blue dye for sentinel lymph node detection in early‐stage cervical cancer: A systematic review and meta‐analysis
Source: Cancer Rep (Hoboken). 2021 May 11;5(1):e1401. doi: 10.1002/cnr2.1401 (PMC8789613; doi:10.1002/cnr2.1401)
Supplement: Supplementary file 8 — Table S1. Detailed study characteristics [file CNR2-5-e1401-s002.doc]

**Table S1. Detailed study characteristics**

| **First author, year** | **Median age, years (range)** | | **Median BMI, kg/m^2^ (range)** | | **Median detected SLNs per patient, n (range)** | | **Surgical procedure** | **Pre-operative 99mTc imaging** | **NIR fluorescence imaging system (manufacturer)** | **Details of injection method**  **Concentration, volume, type** | | | **Pathological assessment SLNs** |
| --- | --- | --- | --- | --- | --- | --- | --- | --- | --- | --- | --- | --- | --- |
|  | **ICG** | **99mTc + BD** | **ICG** | **99mTc + BD** | **ICG** | **99mTc + BD** |  |  |  | **ICG** | **99mTc** | **BD** |  |
| Buda, 2016 (1)^24^ | NA | NA | NA | NA | NA | NA | Laparoscopy or laparotomy^a^ | LSG or SPECT-CT^b^ | SPIES Full HD D-Light P (Karl Storz) | 1.25 mg/mL, 4mL in total | NA | NA | FS: not performed  Ultrastaging: yes |
| Buda, 2018 ^25^ | 42 (28-68) | 46 (29-71) | 22 (16–34) | 24 (17–35) | 3 (2-15) | 2 (0-5) | Laparoscopy (all) | LSG (SPECT-CT if required) | Full HD Image 1S ICG camera (Karl Storz)  PinPoint S1 (Novadaq) | 1.25mg/mL, 4mL in total | 12 MBq in 0.2-0.3mL, nanocolloid | 4mL, 1% methylene blue | FS: yes  Ultrastaging: yes |
| Buda, 2016 (2)^26^ | 42 (25-75) | 47 (26-85) | 23.7 (16–35) | 24.0 (18–41) | 3 (1-15) | 2 (0-7) | Laparoscopy (all) | LSG (SPECT-CT if required) | SPIES Full HD Image 1S H3-Z FI camera (Karl Storz) | 1.25 mg/mL, 4-5mL in total | 7.4-11.1 MBq, volume NA, nanocolloid | 4mL, 1% methylene blue | FS: no information  Ultrastaging: yes |
| Di Martino, 2017 ^27^ | 46 (25–72) | 49 (26–77) | 24.2 (18.4–35.2) | 24.1 (17.4–41.5) | 3 (1-15) | 2 (0-7) | Laparoscopy (all) | LSG (SPECT-CT if required) | SPIES Full HD Image 1S H3-Z FI camera (Karl Storz) | 1.25 mg/mL, 4-5mL in total | 7.4-11.1 MBq, volume NA, nanocolloid | 4mL, 1% methylene blue | FS: yes  Ultrastaging: yes |
| Imboden, 2015 ^28^ | 43.4* | 47* | NA | NA | 3,7* (NA) | 2,1* (NA) | Laparoscopy (all) | SPECT-CT | ‘NIR fluorescent optic device’ (Karl Storz) | 1.25 mg/mL, 8-10mL | 120 MBq, volume NA, type NA | 5mL patent blue | FS: yes  Ultrastaging: yes |
| Salvo, 2017 ^29^ | NA | NA | NA | NA | NA | NA | Laparotomy, laparoscopy or robot-assisted laparoscopy^a^ | NA | NA | NA | NA | NA | FS: Not routinely performed^d^  Ultrastaging: yes |
| Snyman, 2018 ^30^ | NA | NA | NA | NA | NA | NA | Laparotomy or laparoscopy^a^ | LSG | NA | NA | NA | 2mL methylene blue | FS: yes  Ultrastaging: yes |
| Soergel, 2018 ^31^ | 50.7* | SI | NA | NA | 5,6* | 3,2* | Laparoscopy (all) | SPECT-CT^c^ | D-Light P (Karl Storz) | 1.25mg/mL, 4mL in total | 10 MBq, in four doses of 2.5 MBq in 0.3 mL, nanocolloid | 4mL patent blue | FS: yes  Ultrastaging: no information |

ICG, Indocyanine Green; 99mTc, Technetium-99m nanocolloid; BD, Blue Dye; SLN, Sentinel Lymph Node; LSG, lymphoscintigraphy; SPECT-CT, single photon emission computed tomography-computed tomography; NA, Not Available. SI, Same as Intervention group; FS, Frozen Section. *Mean instead of median (no standard deviation provided).
^a^ Number of cervical cancer patients per surgical procedure is not described; ^b^ Protocol not clear on modality of pre-operative imaging; ^c^ 28/33 patients received SPECT-CT, in five cases SPECT-CT was not possible due to refusal by the patient or technical problems; ^d^ SLNs were not routinely sent for frozen section analysis unless there was evidence of metastatic disease on gross inspection.
